# Gallstone disease classification using SLOA-optimized CatBoost classifier with explainable AI

**DOI:** 10.1371/journal.pone.0342945

**Published:** 2026-06-01

**Authors:** Prosenjit Das, Md. Ayaj Uddin Khan, Proshenjit Sarker, Abdullah-Al Nahid

**Affiliations:** 1 Electronics and Communication Engineering Discipline, Khulna University, Khulna, Bangladesh; 2 Department of Electrical and Electronic Engineering, Bangladesh Army University of Science and Technology Khulna, Khulna, Bangladesh; Federal University of Technology - Parana, BRAZIL

## Abstract

Gallstones are small stones that form in the gallbladder. Around 80% of individuals with gallstones do not present any symptoms. Despite the high accuracy of image-based machine learning (ML) models in the detection of gallstones, less research has been carried out regarding tabular data. This paper has discussed a publicly available tabular dataset in order to study some predictive models to determine the presence of gallstones. We have employed the Catboost (CB) classifier model and the Sea Lion Optimization Algorithm (SLOA) in our study. In this project, the primary methods that are explored include CatBoost with cross-validation and CB optimized using the SLOA with cross-validation. The CB model techniques using 5-fold cross-validation have attained a mean accuracy of 79.58%, a mean F1-score of 79.01%, a mean precision of 80.91%, and a mean recall of 77.36% using a total of 38 features. In particular, fold-1 has attained an accuracy of 86.46%, F1-score 85.39%, precision of 88.37%, and recall of 82.61% among the 5-fold cross-validation of the CB model. In addition, the SLOA_CB model of 5-fold cross-validation has achieved a mean accuracy of 80.42%, a mean F1-score of 79.94%, a mean precision of 81.97%, and a mean recall of 77.76%, using the 19 selected features. In this case, fold-4 has attained an accuracy of 87.50%, F1-score, precision, and recall of 87.23%, which implies that the classification performance is balanced. Lastly, SHAP, LIME, and DiCE have been applied to the model explainability, and the most influential features in all cases, regarding the prediction of gallstone disease, are C-Reactive Protein (CRP) and Vitamin D.

## 1. Introduction

Gallstone (GS), medically known as cholelithiasis, is represented by such a solid stone in the gallbladder or bile ducts that is formed by the supersaturated and precipitated components of bile. The formation of this stone is the effect of an imbalance of the constituents of bile, i.e., mainly cholesterol, bile salts, and bilirubin, that become precipitated as hard lumps. The gallstones vary widely in size, varying between very small granular form and forms of more than several centimeters, in their incidence, as single or multiple [1]. Gallstones are widespread, with 10–15% of the people in the Western world being affected, though there is a higher rate among adults, women, and even in some ethnic groups such as the Native Americans [1]. Gallstones may be painless in many patients, but they can be painful or cause difficulties, and should be addressed medically.

The early detection of gallstones is essential for timely intervention and reducing the burden on both patients and healthcare systems [2,3]. Gallstones, which form in the gallbladder, can lead to serious complications like cholecystitis and pancreatitis if not detected and treated promptly. Early diagnosis enables less invasive treatments, preventing costly emergency procedures. The medical data produced by sensors on the body and the IoT devices including heart rate and glucose monitoring devices, has a tendency of generating huge amounts of physiological data. The need to manage such datasets ought to have safe and scalable designs that guarantee privacy and trustworthy data processing in healthcare settings [4]. Machine learning (ML) has shown promise in detecting diseases like gallstones by analyzing complex datasets and uncovering patterns that clinicians may overlook [5,6]. This study explores the use of the Sea Lion Optimization Algorithm (SLOA) combined with the CatBoost classifier for the early detection of gallstones, focusing on tabular data, which remains less explored compared to image-based methods.

Deep learning, especially Convolutional Neural Networks (CNNs) appeared to have a promising future of early detection of several medical conditions based on ultrasound and histopathological images, shown by a recent study on maternal health and tissue analysis [7]. Most previous research has concentrated on image-based techniques for gallstone detection, using methods such as ultrasound, CT scans, and MRI. For example, Bozdog et al. achieved 94.4% accuracy with Content-Based Image Retrieval (CBIR) [8], while Wang et al. reported a value of 0.995 with an ultrasound-based model for an area under the curve (AUC) [9]. Pang et al. used YOLO on CT scan images, achieving 86.5% accuracy [10], and Obaid et al. and Hong et al. reached accuracies of 98.35% and 96.33%, respectively, with image-based approaches [11,12]. However, these models face challenges, such as high computational costs and slow processing speeds due to the large dataset sizes generated by high-resolution medical images [13]. Additionally, image-based models often lack interpretability, making it difficult for clinicians to understand the reasoning behind predictions—a key limitation in clinical decision-making [14–18].

In contrast, tabular data-based models, which use structured clinical test results, offer a more computationally efficient alternative [19]. These models are faster to process and provide clearer insights into the factors influencing predictions, improving interpretability. Esen et al. predicted the presence of gallstones with 85.42% accuracy using a Gradient Boosting Classifier on a tabular dataset [20]. Similarly, Li et al. developed a machine learning framework to predict gallstone disease using clinical data from the UCI dataset. They employed principal component analysis (PCA) and multi-strategy feature selection with a stacking ensemble model combining Random Forest, XGBoost, and Support Vector Machine. Their model achieved a strong AUC of 0.9102 and accuracy of 81.25% [21]. Tabular datasets often include many features, some of which represent costly clinical tests. Including irrelevant or redundant features can lead to overfitting and computational burden [22,23]. Chakraborty and Mukherjee developed a hybrid machine learning framework combining Adaptive LASSO, Bayesian Additive Regression Trees, and logistic regression with differential equation-informed interactions to improve gallstone risk prediction. The model identified key predictors like C-reactive protein and visceral fat area—while revealing significant interactions between CRP and hemoglobin, and Vitamin D and hyperlipidemia—outperforming traditional models in accuracy [24]. Even though progress has been made in the past research, there are also a number of key limitations. Most of the current methods are based on highly complicated machine learning models and use a great number of clinical features, which can make them less realistic when applied in a medical environment [20]. Moreover, little effort has been made to examine misclassified cases, and it is hard to know the circumstances in which the models do not work. The lack of transparency has been another significant issue, since most of the former models that were developed tend to act as black box and fail to give any explanations in their predictions [21,24]. As a result, clinicians are in most cases unable to understand why a given case is predicted to be either gallstone positive or negative. This study addresses these issues by using the Sea Lion Optimization Algorithm to reduce the dataset’s dimensionality, choosing only the most significant features, and fine-tuning the hyperparameters of the CatBoost classifier for optimal performance.

To tackle the “black-box” aspect of machine learning models, this research incorporates techniques from Explainable AI (XAI). Shapley Additive Explanations (SHAP) will analyze feature contributions, revealing which factors most influence the model’s predictions. In addition, the research has used Local Interpretable Model-agnostic Explanations (LIME). The LIME method is an approximation method that estimates any black box machine learning model with a local explainable model to explain the individual prediction. Diverse Counterfactual Explanations (DiCE) will offer alternative explanations, helping clinicians understand how changes in input features could affect the outcome. By combining feature selection, hyperparameter tuning, and explainable AI, this study aims to develop a model that is not only accurate and efficient, but also interpretable—thus enhancing its clinical utility. The use of tabular data, alongside these techniques, will contribute to more accurate, cost-effective, and transparent early detection of gallstones, improving decision-making and patient outcomes in clinical settings.

Our study focuses on the deployment of metaheuristic algorithms, custom k-fold cross-validation, and explainable AI methods, including SHAP, DiCE, and LIME, unlike previous studies that did not combine all methods into a single study. In this research, our main objectives are:

- Firstly, we have applied the CB classifier with cross-validation.
- Secondly, we have deployed a metaheuristic algorithm to reduce features and hyper-tune the classification model.
- Third, we have analyzed the importance of the features using SHAP.
- Lastly, we have utilized LIME and DiCE for misclassification analysis.

The rest of the paper is divided into 4 sections. Section 2 provides Materials and methods, Section 3 Results, Section 4 Discussion and Section 5 Conclusion.

## 2. Materials and methods

**Fig 1:**
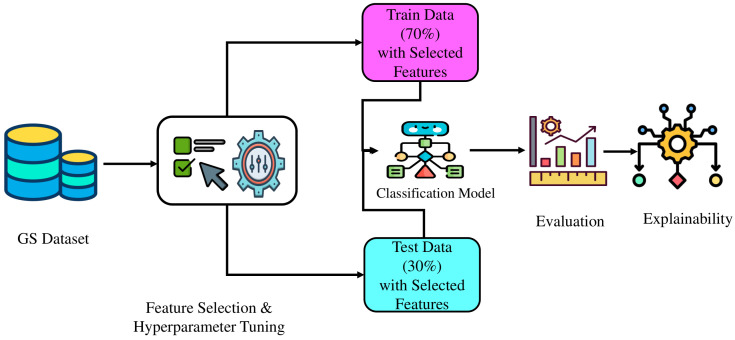
Sequential steps of the proposed methodology.

This research has classified patients with positive gallstones (GP) and negative gallstones (GN) according to the GS dataset. GP and GN patients have been appropriately labeled in this dataset. The task has been started with data splitting in a suitable ratio, in which 70% data has been used in training cases and 30% data has been applied to test cases, then we will select a classification model. After selecting the classification model, we will perform optimization for selecting features and hyper-tuning for the classifier parameters. The optimum hyper-parameter values and a selection of features from the training and testing sets will then be used to create the optimized model, which will perform the final classification. In this case, we will apply the k-fold cross-validation technique. Finally, SHAP, LIME, and DiCE have been applied for explainability purposes. Fig 1 shows the overall workflow of this research work.

### 2.1 Dataset

In this study, the GS dataset was utilized, which was provided by the Internal Medicine Outpatient Clinic of Ankara VM Medical Park Hospital. The dataset has a total of 319 individual samples. In this dataset, 161 individuals have been diagnosed with GP (0), and 158 individuals have not been diagnosed; they are GN (1) individuals. This dataset can be found in UC Irvine Machine Learning Repository and URL: https://archive.ics.uci.edu/dataset/1150/gallstone-1, accessed on: 02 August, 2025. The dataset was originally introduced by Esen et al [20]. The authors have mentioned that, the dataset was approved by the Ethics Committee of Ankara City Hospital Medicine (E2-23–4632) [20]. This dataset includes 38 features, including demographic, bioimpedance, and laboratory data. Table 1 shows the description of the dataset, including the minimum, maximum, and mean values.

**Table 1:** Summary statistics for features of GP and GN individuals.

Feature	Index	Data Completeness (%)	GP (0)			GN (1)		
			Avg	Max	Min	Avg	Max	Min
Age (F1)	0	100	56.7	89.0	24.0	59.3	86.0	34.0
Gender (F2)	1	100	0.48	1.00	0.00	0.51	1.00	0.00
Comorbidity (F3)	2	100	1.2	5.0	0.0	1.6	6.0	0.0
Coronary Artery Disease (CAD) (F4)	3	100	0.15	1.0	0.0	0.30	1.0	0.0
Hypothyroidism (F5)	4	100	0.12	1.0	0.0	0.28	1.0	0.0
Hyperlipidemia (F6)	5	100	0.35	1.0	0.0	0.42	1.0	0.0
Diabetes Mellitus (DM) (F7)	6	100	0.24	1.0	0.0	0.31	1.0	0.0
Height (F8)	7	100	165.3	190.0	145.0	163.7	185.0	140.0
Weight (F9)	8	100	71.2	103.0	45.0	75.6	115.0	49.0
BMI (F10)	9	100	26.1	34.8	18.4	28.3	38.5	19.2
Total Body Water (TBW) (F11)	10	100	40.2	60.1	28.3	42.0	62.5	29.1
Extracellular Water (ECW) (F12)	11	100	15.2	21.1	9.5	16.0	22.3	10.1
Intracellular Water (ICW) (F13)	12	100	25.0	39.0	18.0	26.0	40.2	18.5
Extracellular Fluid (ECF) (F14)	13	100	19.1	28.3	14.4	19.8	29.2	15.1
Total Body Fat Ratio (TBFR) (%) (F15)	14	100	25.7	38.5	12.6	28.9	41.7	14.3
Lean Mass (LM) (%) (F16)	15	100	62.4	78.1	49.3	59.2	75.3	48.0
Body Protein Content (Protein) (%) (F17)	16	100	17.2	22.0	14.1	16.4	21.0	13.9
Visceral Fat Rating (VFR) (F18)	17	100	6.3	13.0	2.0	8.2	17.0	3.0
Bone Mass (BM) (F19)	18	100	2.4	3.3	1.8	2.3	3.1	1.7
Muscle Mass (MM) (F20)	19	100	46.8	65.3	32.1	45.0	63.7	30.4
Obesity (%) (F21)	20	100	23.2	36.1	11.0	26.7	39.5	13.3
Total Fat Content (TFC) (F22)	21	100	19.3	29.5	8.6	21.7	32.4	10.2
Visceral Fat Area (VFA) (F23)	22	100	85.4	148.6	39.2	96.7	165.3	45.8
Visceral Muscle Area (VMA) (Kg) (F24)	23	100	23.5	32.2	16.8	22.4	30.9	15.4
Hepatic Fat Accumulation (HFA) (F25)	24	100	4.3	7.9	1.2	5.6	9.2	1.6
Glucose (F26)	25	100	92.7	130.0	74.0	98.3	145.0	76.0
Total Cholesterol (TC) (F27)	26	100	185.2	235.0	143.0	192.6	247.0	150.0
Low Density Lipoprotein (LDL) (F28)	27	100	112.4	165.0	74.0	117.3	172.0	78.0
High Density Lipoprotein (HDL) (F29)	28	100	50.1	65.3	39.0	47.6	61.2	35.5
Triglyceride (F30)	29	100	145.6	210.0	85.0	153.8	228.0	92.0
Aspartat Aminotransferaz (AST) (F31)	30	100	22.7	43.0	12.0	26.1	48.0	14.0
Alanin Aminotransferaz (ALT) (F32)	31	100	24.3	50.0	13.0	27.9	54.0	15.0
Alkaline Phosphatase (ALP) (F33)	32	100	78.2	110.0	52.0	82.5	115.0	55.0
Creatinine (F34)	33	100	0.86	1.2	0.6	0.92	1.4	0.7
Glomerular Filtration Rate (GFR) (F35)	34	100	92.1	120.0	65.0	89.4	118.0	62.0
C-Reactive Protein (CRP) (F36)	35	100	1.8	4.5	0.3	2.6	5.8	0.4
Hemoglobin (HGB) (F37)	36	100	13.8	16.4	11.2	13.4	16.0	10.8
Vitamin D (F38)	37	100	24.5	38.0	12.4	21.7	35.6	11.1

### 2.2 Classification method

In this study, we have used the CatBoost (CB) classifier. CB is a high-performance gradient-boosting algorithm designed to handle categorical features efficiently [25]. Developed by Yandex [26], it builds decision trees sequentially, each tree learning from the previous one to improve prediction accuracy [27]. Unlike other gradient-boosting algorithms, CB automatically handles categorical data without requiring manual preprocessing like one-hot encoding [25]. This unique feature allows it to efficiently process mixed datasets containing both categorical and numerical variables [28].

The algorithm uses ordered boosting [29], which reduces overfitting by performing multiple permutations of the training data, thus preventing target leakage [27]. CB excels when categorical data is present, but its performance decreases without it [30]. The method leverages modified target-based statistics to handle categorical variables, saving memory and computation time compared to traditional encoding techniques [27]. It also uses oblivious decision trees, where the same split criterion is applied across each tree level, making the trees balanced and reducing overfitting [31]. The CB includes built-in cross-validation and class weighting features, allowing for better model evaluation and handling of class imbalance. It also supports GPU acceleration, speeding up training on large datasets [25].

For this study, the hyperparameters utilized for the CB classifier include the iterations, l2_leaf_regularization, learning_rate, and depth. The boundary condition established for the iterations range from 100 to 300, with the L2 egularization varying from 1 to 10, etc. Table 2 provides a detailed breakdown of all hyperparameter ranges and types.

**Table 2:** Hyperparameter limits for the CB classifier.

Hyperparameter	Type	Range
Iterations	Integer	100–300
L2_Leaf_Regularization	Float	1–10
Learning Rate	Float	0.001 to 0.2
Depth	Integer	4–8

### 2.3 Justification of choosing CB

Table 3 presents the F1 scores of five various classifiers that have been used to determine the gallstone status. CatBoost classifier has the highest F1 score of 82.98%, which is the highest balance between precision and recall, as the rest of the evaluated models have lower F1 scores. Though other classifiers like XGBoost, Gradient Boosting, LightGBM, and AdaBoost classifiers are marginally higher in precision, their low recall resulted in low F1 scores (81.31%, 82.60%, 80.00%, and 77.77% respectively). As the F1 score takes both the false positive and false negative into consideration, for this reason, CatBoost has been chosen because it gives the best predictive performance.

**Table 3:** Comparison of classifiers based on F1 score (%).

Classifier	F1 Score (%)
CatBoost	82.97
XGBoost	81.31
Gradient Boosting	82.60
LightGBM	80.00
AdaBoost	77.77

### 2.4 Sea Lion Optimization Algorithm

The Sea Lion Optimization Algorithm (SLOA) method draws inspiration from the hunting behavior of sea lions, known for their intelligence, hierarchical social structures, and exceptional prey-tracking abilities [32]. Sea lions use their whiskers to sense the size, shape, and position of prey, detecting waves created by the prey [33]. They adjust their orientation to track and encircle their target. Their strategy—moving efficiently and often in groups—includes trapping prey at the edges of bait balls. SLOA mathematically models these behaviors to optimize solutions.

Sea lions live in large, hierarchical colonies and navigate through subgroups based on age, sex, and role. They primarily hunt using their whiskers, which detect prey by sensing the waves left behind. These whiskers, extending up to 30 cm, help sea lions identify prey in murky waters where vision is limited. Sea lions also communicate through vocalizations, coordinating their movements and encircling prey. The leader directs the group to the best hunting spots.

Tracking and Detection: Sea lions track prey by detecting the direction, size, and position using their whiskers. The mathematical model for this is outlined in Eq. (1) [34], where the sea lion’s position is calculated in relation to the prey. The sea lion’s position updates each iteration to bring it closer to the prey, as shown in Eq. (2) [34]. A random vector is used to explore the solution space for optimization.


$$\begin{array}{c} \vec{Dist}=\left|\vec{2B}\cdot \vec{P}(t)-\vec{SL}(t)\right| \end{array}$$ (1)

$$\begin{array}{c} \vec{SL}(t+1)=\vec{P}(t)-\vec{Dist}\cdot \vec{C} \end{array}$$ (2)

The vector $\vec{Dist}$ denotes the distance between the target prey and the sea lion, with the position vectors of the prey and sea lion represented as $\vec{P}(t)$ and $\vec{SL}(t)$, respectively. The current iteration, denoted as *t*, uses a random vector $\vec{B}$ within [0, 1], scaled by 2 to expand the search space and assist agents in finding optimal solutions; (*t* + 1) represents the nex*t* iteration, and $\vec{C}$ decreases linearly from 2 to 0 over the course of iterations, which forces the sea lion’s leader to move toward and surround the prey.

Vocalization Phase: Sea lions use vocalizations to communicate, particularly when hunting in groups [35]. Their calls travel fourfold more rapidly underwater compared to in the atmosphere [36]. This difference in sound speed allows them to coordinate with others both underwater and on land. This condition is mathematically represented in Eqs. (3)–(5) [34], which account for sound propagation through different media.


$$\begin{array}{c} {\vec{SP}}_{leader}=\left|\frac{{\vec{V}}_{1}(1+{\vec{V}}_{2})}{{\vec{V}}_{2}}\right| \end{array}$$ (3)

$$\begin{array}{c} {\vec{V}}_{1}=\sin\theta \end{array}$$ (4)

$$\begin{array}{c} {\vec{V}}_{2}=\sin\varphi \end{array}$$ (5)

The vector $\vec{{SP}_{leader}}$ represents the sea lion leader’s speed of sound, with ${\vec{V}}_{1}$ and ${\vec{V}}_{2}$ indicating the speed of sound in water and air, respectively; when the sea lion emits a sound, it reflects in air (to communicate with members on the shore) and refracts in water (to signal underwater members), with the first case denoted by (sinθ) and the second by (sinφ).

Attacking Phase (Exploitation): In this phase, the sea lion leader guides the group toward the prey. This behavior is modeled in two stages:

Dwindling Encircling: The sea lion reduces the distance to the prey with each iteration, moving closer as described in Eq. (2) [34].

Circle Updating: Sea lions encircle their prey by swimming in a circular path. This is mathematically represented by Eq. (6) [34], where sea lions begin hunting the prey at the edge of the bait ball.


$$\begin{array}{c} \vec{SL}(t+1)=\left|\vec{P}(t)-\vec{SL}(t)\right|\cdot \cos(2\pi m)+\vec{P}(t) \end{array}$$ (6)

The expression $\left|\vec{P}(t)-\vec{SL}(t)\right|$ represents the distance between the optimal solution (target prey) and the search agent (sea lion), with || denoting the absolute value and *m* as a random number in [−1, 1]; this captures the sea lion’s behavior of swimming in a circular path around the prey (bait ball) to target those at the edge, mathematically expressed by (cos(2πm)).

Exploration Phase: In the Exploration phase, sea lions search for prey by swimming in a random zigzag pattern, guided by their whiskers. The SLOA models this behavior using vectors, allowing agents to explore diverse solutions. When the random value exceeds one or falls below negative one, the sea lion moves away from the prey, prompting the agent to search for new prey. This behavior is mathematically represented in Eqs. (7) and (8) [34], where agents update their positions based on a randomly selected sea lion. This randomness enables the SLO algorithm to conduct a global search, increasing the likelihood of finding the optimal solution.


$$\begin{array}{c} \vec{Dist}=\left|2B\cdot {\vec{SL}}_{rnd}(t)-\vec{SL}(t)\right| \end{array}$$ (7)

$$\begin{array}{c} \vec{SL}(t+1)={\vec{SL}}_{rnd}(t)-\vec{Dist}\cdot \vec{C} \end{array}$$ (8)

The vector ${\vec{SL}}_{rnd}(t)$ represents a randomly selected sea lion from the current population.

The SLOA begins with random solutions and iteratively updates positions based on the best solution or a randomly chosen agent. Parameter C, which decreases from 2 to 0, controls the balance between exploration and exploitation. The algorithm continues until a stopping criterion is met.

We used the SLOA algorithm to fine-tune the hyperparameters of the CB classifier and perform feature selection. To ensure reproducibility, we set the seed to 10, the random state to 42, and optimized for 50 epochs with a population of 30. The search space includes four hyperparameters and a binary feature selection vector that identifies potential features. Random initialization of the population occurs within the defined boundaries. Our goal is to maximize the F1-score on the training set. Convergence occurs when either 50 epochs are reached or the global best fitness value stabilizes.

### 2.5 Justification of choosing SLOA

In our study, we have chosen a metaheuristic-based SLOA algorithm because it can balance exploration and exploitation during the search process [34]. For instance, Sea Lion Optimization has recently been employed with deep learning models for automated detection and classification of meningioma tumors from MRI images where SLO has improved the process of extracting features and classifying the tumors resulting in higher accurate predictions [37].

Table 4 shows performance comparison between various optimization algorithms with the CatBoost classifier. With the best accuracy of 81.25% in the testing and identifying only 19 features, Sea Lion Optimization (SLOA) is the most suitable among the considered methods. The Aquila Optimizer (AO) has the same test accuracy (81.25%); however, it has used 38 features, which is lower in terms of feature selection efficiency. By comparison, Golden Jackal Optimization (GJO) only chose 5 features, although its test accuracy is reduced to 57.29%, indicating inadequate predictive ability. In addition, the Honey Badger Algorithm (HBA) used 7 features and has a 69.79% accuracy which remains less than SLOA.

**Table 4:** Performance comparison using different optimization algorithms.

Optimization Algorithm	Classifier	Test Accuracy (%)	Selected Features
Sea Lion Optimization	CatBoost	81.25	19
Aquila Optimizer	CatBoost	81.25	38
Golden Jackal Optimization	CatBoost	57.29	5
Honey Badger Algorithm	CatBoost	69.79	7

Hence, keeping in mind both classification performance and reduction of features, SLOA offers a more optimal balance between the accuracy and the number of selected features. This results in SLOA being a more suitable optimization algorithm for the proposed gallstone disease prediction.

### 2.6 Custom 5-fold SLOA_CB optimization

A custom 5-split evaluation strategy has been applied. The dataset has been randomly shuffled and divided into 70% training and 30% testing subsets for each split. Five iterations of rotation of the test set through the dataset were carried out, and every sample was tested at least once. The approach will enable a detailed and consistent assessment of the performance of the model on various subsets of the data. Data shuffling has been conducted with a fixed random state of 42, and the optimizer seed is 10 in order to guarantee the reproducibility of the results. A metaheuristic optimization that uses SLOA has been performed in each fold to optimize both hyperparameters of CB and also to choose the most relevant features. The hyperparameters, being iterations, learning rate, depth, and L2 leaf regularization, are encoded in each solution, and the feature selection is a binary vector. The optimizer will be run over 50 epochs, and the population size will be 30 candidate solutions. The objective function will be used to assess the training F1 score on the chosen features to direct the search towards the optimal performance. The search space is stipulated as repetitions of 100–300, learning rate of 0.001–0.2, depth of 4–8, L2 leaf regularization of 1–10, and binary selection of all the available features. When optimized, the CB model will be trained using the chosen features and the performance compared on the training and test sets.

### 2.7 Evaluation criteria

This section evaluates the model’s performance using the following metrics: Accuracy, F1-score, Precision, Recall, Confusion matrix, and ROC curve. The evaluation metrics are calculated using the following formulas:


$$\begin{array}{c} \text{Accuracy}=\frac{TP+TN}{TP+TN+FP+FN} \end{array}$$ (9)

where TP, TN, FP, and FN represents True Positive, True Negative, False Positive, and False Negative, respectively.


$$\begin{array}{c} \text{Precision}=\frac{TP}{TP+FP} \end{array}$$ (10)

$$\begin{array}{c} \text{Recall}=\frac{TP}{TP+FN} \end{array}$$ (11)

$$\begin{array}{c} \text{F1-Score}=\frac{2\times \text{Precision}\times \text{Recall}}{\text{Precision}+\text{Recall}} \end{array}$$ (12)

### 2.8 SHAP

SHAP is a widely used tool in XAI that interprets machine learning model predictions by quantifying the contribution of each feature. Based on Shapley values from cooperative game theory, SHAP provides a clear importance score for each feature in a given prediction [38,39]. Studies have demonstrated SHAP’s effectiveness in various domains, such as liver disease classification, where it helped explain model outcomes, and in feature selection tasks, where it achieved accuracy levels up to 99.55% [40,41]. In breast cancer classification, SHAP assessed feature quality and contributed significantly to model explainability [42]. Given its superior performance compared to other XAI tools, SHAP was chosen for this study. We divided our analysis into two parts: SHAP evaluation with and without MHA mechanisms.

SHAP not only identifies the most impactful features globally but also explains individual predictions. By analyzing mean SHAP values and visualizing them through bar plots, we assessed overall model performance. Additionally, we examined individual predictions by evaluating the SHAP values for each feature, determining whether a prediction leans toward the positive or negative class. SHAP’s ability to interpret model predictions, both globally and locally, makes it an invaluable tool in the field of Explainable Artificial Intelligence, particularly in complex models like Gradient Boosting, XGBoost, Random Forests, and Neural Networks [43].

### 2.9 LIME

LIME is a technique used to interpret the predictions of machine learning models [44]. In addition, LIME is a model-agnostic method that explains individual predictions by creating a simple surrogate model around a specific instance [44]. It perturbs the input data locally and uses the surrogate to reveal which features most influenced the black-box model’s decision [44]. The explanations provided by LIME for each observation *x* are obtained as follows [45]:


$$\begin{array}{c} \xi (x)=\text{arg}\min_{g\in G}\;ℒ(f,g,\pi_{x})+\Omega (g) \end{array}$$ (13)

where *G* is a class of potentially interpretable models, g∈G is a candidate explanation model, and *f* is the original black-box model being explained. $\pi_{x}$ is a proximity measure that quantifies how close an instance is to *x*, and Ω(g) measures the complexity of the explanation model *g*.

The term $ℒ(f,g,\pi_{x})$ quantifies the unfaithfulness of *g* in approximating *f* in the locality defined by $\pi_{x}$. Thus, the objective of LIME is to minimize the unfaithfulness $ℒ(f,g,\pi_{x})$ while keepin*g* the complexity Ω(g) sufficiently low so that the explanation remains interpretable by humans.

### 2.10 DiCE

DiCE is an approach based on counterfactual reasoning that is used to create hypothetical scenarios for machine learning models. It shows how input features must change to modify the model’s prediction. The technique generates a variety of plausible counterfactuals, providing users with several options to reach the desired result [46]. DiCE allows for adjustment of parameters to control the diversity and proximity of these counterfactuals, ensuring practical alternatives that are close to the initial input.

DiCE’s optimization objective is outlined as follows [46]:


$$\begin{array}{c} C(x)=\text{arg}\min_{c_{1},\ldots ,c_{k}}\left[\frac{1}{k}\sum_{i=1}^{k}\text{yloss}(f(c_{i}),y)+\lambda_{1}\frac{1}{k}\sum_{i=1}^{k}\text{dist}(c_{i},x)-\lambda_{2}\cdot \text{dpp\_diversity}(c_{1},\ldots ,c_{k})\right] \end{array}$$ (14)

Here, $\text{yloss}(f(c_{i}),y)$ represents the classification loss, which drives the counterfactuals toward the target class. The proximity term dist(*c*_*i*_, *x*) ensures that the generated counterfactuals remain close to the original input, retaining realism. The dpp_diversity term uses Determinantal Point Processes to promote heterogeneity across the counterfactuals. The hyperparameters $\lambda_{1}$ and $\lambda_{2}$ regulate the trade-off between proximity and diversity. DiCE offers a model-agnostic approach, generating counterfactual examples that assist users in understanding how changes to input features influence model predictions. By optimizing the above objective, DiCE provides actionable insights and multiple viable options to alter the model’s output.

### 2.11 Experimental setup

Table 5 provides a summary of the experimental environment and configuration. The fixed random seeds and a predetermined SLOA setting were used to obtain reproducibility.

**Table 5:** Software environment and configuration used in the experiments.

Category	Parameter	Value
Software Stack	Python	3.12.12
	NumPy / Pandas	1.26.0 / 2.2.2
	XAI Frameworks (SHAP / LIME / DiCE)	0.51.0 / 0.2.0.1 / 0.12
Optimization	Mealpy	3.0.3
Reproducibility	Random State/Seed	42
	SLOA Seed	10
SLOA Configuration	Epoch / Population	50 / 30

## 3. Results

In this section, the experimental results of our study have been presented. In this study, we have described our results in two parts: Performance evaluation using custom k-fold validation with CB classifier, Performance evaluation using custom k-fold validation with SLOA_CB classifier.

### 3.1 Performance evaluation using custom k-fold validation with CB classifier

In this case, we have explained our results into 5 categories: Performance evaluation, Confusion matrix analysis, ROC curve analysis, SHAP analysis, and DiCE analysis.

#### 3.1.1 Performance evaluation.

The performance of the CB classifier across the five folds has been presented in Table 6. The model has achieved 100% accuracy, F1-score, precision, and recall on the training sets of all folds, indicating a complete fit to the training data. However, the test results have shown variability, with accuracy values ranging between 72.92% and 86.46%. The corresponding mean test accuracy of 79.58%, along with an average F1-score of 79.01%, precision of 80.91%, and recall of 77.36%, has reflected moderate overall performance. Folds 1 and 4 have demonstrated comparatively stronger predictive capability, whereas Fold 3 has exhibited the weakest results, particularly in recall (68.00%). These findings suggest that although the classifier has learned the training data perfectly, its generalization across unseen samples has remained inconsistent and could benefit from further optimization.

**Table 6:** Model results using CB classifier.

Fold	Train				Test			
	Acc. (%)	F1-score. (%)	Pre. (%)	Rec. (%)	Acc. (%)	F1-score (%)	Pre. (%)	Rec. (%)
Fold-1	100	100	100	100	86.46	85.39	88.37	82.61
Fold-2	100	100	100	100	77.08	77.08	75.51	78.72
Fold-3	100	100	100	100	72.92	72.34	77.27	68.00
Fold-4	100	100	100	100	85.42	84.44	88.37	80.85
Fold-5	100	100	100	100	76.04	75.79	75.00	76.60
Mean	100	100	100	100	79.58	79.01	80.91	77.36

#### 3.1.2 Confusion matrix analysis.

**Fig 2:**
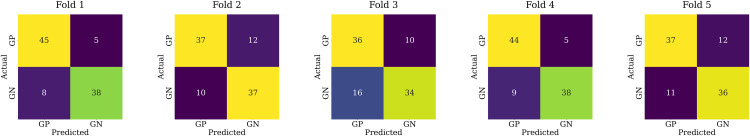
Representation of fold-wise confusion matrix generated by CB.

Fig 2 illustrates the fold-wise confusion matrix obtained using the CB classifier. The CB classifier has correctly classified 45 GP and 38 GN instances in fold 1 with 13 misclassifications. In fold 2, 37 GP and 37 GN have been correctly classified by the CB classifier. The model has created slightly more counts of misclassifications in fold 3, with 36 correct predictions of GP and 34 of GN. Fold 4 has once more shown good performance, 44 GP and 38 GN correctly classified. Finally, fold 5 has demonstrated that 37 GP and 36 GN have been classified by the model. Overall, despite the presence of misclassifications, the classifier has exhibited a relatively stable predictive pattern for both classes (GP and GN) across the folds.

#### 3.1.3 Misclassifications output.

Table 7 presents the fold-wise misclassification outcomes attained using the only CB classifier. For every fold, both the number and percentage of misclassified samples are provided, along with the indices of instances that have been incorrectly predicted by the CB classifier. In fold 1, there are 14 misclassified samples, resulting in a misclassification percentage of 14.58%. Furthermore, fold 2 has recorded 21 misclassifications, equating to 21.88%. In addition, folds 3, 4, and 5 have reported misclassified sample counts of 27, 19, and 20, respectively, with misclassification percentages of 28.12%, 12.50%, and 20.83%.

**Table 7:** Misclassified instances across all folds using the CB classifier.

Fold No.	Number of Misclassified	Misclassification (%)	Misclassified Instances
1	13	13.54	73, 94, 116, 118, 147, 176, 177, 194, 195, 203, 299, 311, 316
2	22	22.92	66, 69, 83, 92, 95, 96, 111, 115, 117, 143, 148, 157, 172, 181, 196, 199, 206, 221, 223, 295, 302, 304
3	26	27.08	8, 52, 81, 91, 105, 110, 128, 141, 153, 156, 178, 192, 200, 207, 215, 216, 225, 227, 243, 251, 281, 291, 297, 309, 313, 315
4	14	14.58	73, 116, 118, 121, 147, 173, 174, 176, 177, 185, 194, 299, 317, 318
5	16	23.96	16, 30, 66, 69, 92, 94, 96, 115, 117, 119, 143, 148, 163, 181, 195, 196, 203, 221, 226, 295, 304, 311, 316

#### 3.1.4 ROC curve analysis.

**Fig 3:**
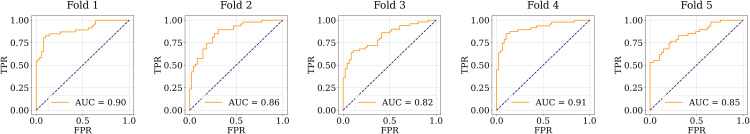
Visualization of ROC curve across folds with CB.

Fig 3 shows the ROC curves of the five folds on the CB classifier. The values of the AUC are between 0.82 and 0.91, which is a good and consistent classification performance across folds. Fold 4 has the highest AUC (0.91), whereas fold 3 has the lowest performance with an AUC of 0.82. Overall, the curves show reliable discriminative ability of the CB model throughout cross-validation.

#### 3.1.5 SHAP analysis.

**Fig 4:**
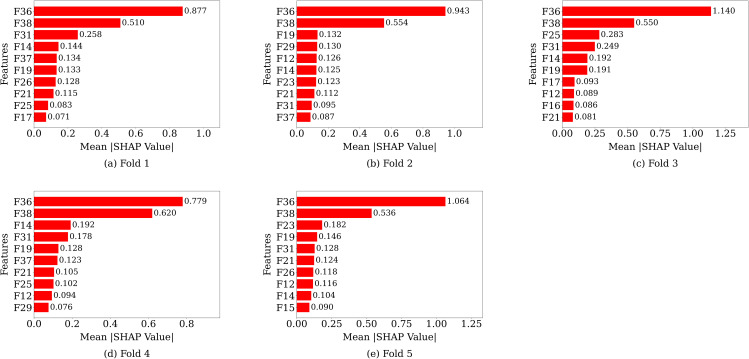
Mean absolute SHAP value of top 10 features for each fold using CB classifier.

Fig 4 demonstrates the mean absolute SHAP value of the top 10 features for each fold using only the CB classifier. In this figure, the horizontal bars indicate how much each feature contributes to the model’s predictions, with longer bars indicating greater importance. In all folds, features F36 and F38 are the most influential compared to the other features. In this case, the highest mean SHAP value was found in fold 3 for F36, with a value of 1.140. Additionally, the lowest SHAP value has been observed in fold 4 at 0.779, which, despite being the minimum overall, represents the highest feature importance within that specific fold. Finally, we can conclude that the features F36 and F38 are the most important features for the prediction.

#### 3.1.6 LIME analysis.

Table 8 presents the LIME explanations for misclassified instances generated by the CB classifier model. Although the model has generated several misclassifications, only two misclassified instances (indices 73 and 176) have been selected for detailed LIME explanations. Class 1 (0.6660) has been observed to have a higher probability of Index 73 (actual 0 – predicted 1), and there is a strong feature influence towards the wrong class. Similarly, the index 176 (actual 1 – predicted 0) has shown a greater Class 0 probability (0.6409), resulting in misclassifications. These LIME explanations show contributions of features that have misled the model, and this helps to be able to spot the root cause of the errors.

**Table 8:** LIME explanations for misclassified instances (indices 73 and 176).

Index No.	Actual → Predicted	Prediction Probability Class 0	Prediction Probability Class 1
73	0 → 1	0.3340	0.6660
176	1 → 0	0.6409	0.3591

#### 3.1.7 DiCE analysis.

In all the developed frameworks, CRP and Vitamin D have always been the two most influential features according to feature selection and SHAP analysis. A DiCE explanation has been performed with these specific features to better understand their contribution to individual predictions.

Table 9 displays the five detailed counterfactual (CF) explanations produced by DiCE for the CB classifier and explains the effects of CRP (F36) and Vitamin D (F38) features on the GS status prediction of instance index 73. The actual class of this instance is 0 (GP), but the CB classifier model has predicted class 1 (GN). The CRP has been at 0.63 in all the five counterfactuals, meaning that it has had little effect on the local decision of the model. Conversely, Vitamin D in CF-1 has risen to 27.1 as compared to the initial value of 10.9, and in CF-2 has risen to 43.6 as compared to the initial value of 10.9, and in CF 3 and CF-5 there is a rise to 29.5 and 49.9 respectively, which amounts to a sufficient increase to alter the predicted class of 1 (GN) to 0 (GP) in all cases. These findings indicate that Vitamin D is a substantial and independent influence on the local decision boundary of the CB model, which makes the predictions to correlate with the real state of the gallstones, whereas CRP showed no significant effect.

**Table 9:** Five counterfactual explanations generated using the CB classifier, demonstrating how variations in CRP (F36) and Vitamin D (F38) influence the Gallstone Status prediction for instance index 73.

Counterfactual No.	CRP	Vitamin D	Gallstone Status
1	0.63	10.9 → 27.1	1 → 0
2	0.63	10.9 → 43.6	1 → 0
3	0.63	10.9 → 29.5	1 → 0
4	0.63	10.9 → 49.9	1 → 0
5	0.63	10.9 → 36.6	1 → 0

Table 10 displays five counterfactual explanations generated using DiCE for the CB classifier, demonstrating how variations in the feature values of CRP (F36) and Vitamin D (F38) have influenced the GS status prediction for instance index 176, whose actual class is 1. In all the counterfactuals, CRP has ranged between 0.6 and 17.9 in CF-1, 12.1 in CF-2, 1.2 in CF-3, 34.4 in CF-4, and 17.9 in CF-5. In CF-1, vitamin D has increased to 24.7 compared to 12.7, and in CF-2 to CF-5; it has been 12.7. Such changes have been enough to turn the predicted class of 0 (GP) to 1 (GN) in both cases. The findings indicate that CRP significantly influences the local decision boundary of the CB model but Vitamin D has an effect only in CF-1.

**Table 10:** Five counterfactual explanations generated using DiCE for CB classifier, demonstrating how variations in CRP (F36) and Vitamin D (F38) influence the Gallstone Status prediction for instance index 176.

Counterfactual No.	CRP	Vitamin D	Gallstone Status
1	0.6 → 17.9	12.7 → 24.7	0 → 1
2	0.6 → 12.1	12.7	0 → 1
3	0.6 → 1.2	12.7	0 → 1
4	0.6 → 34.4	12.7	0 → 1
5	0.6 → 17.9	12.7	0 → 1

### 3.2 Performance evaluation using custom k-fold validation with SLOA_CB classifier

In this section, we have explained our results into 6 categories: Optimization results, Performance evaluation, Confusion matrix analysis, ROC curve analysis, SHAP analysis, and DiCE analysis.

#### 3.2.1 Optimization results.

**Fig 5:**
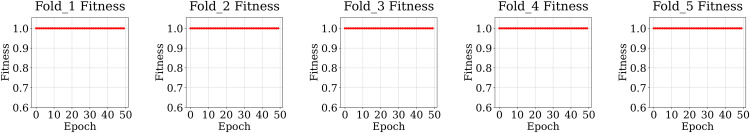
Fitness of each fold.

In this study, we have designed an experimental pipeline with a custom 5-fold cross-validation strategy, where in each fold 70% of the dataset has been applied for training and the remaining 30% for testing. For each fold, we have used the SLOA for hyperparameter tuning and feature selection purposes. The optimization objective is to maximize the F1-score on the training set. In each fold, the SLOA has successfully attained a fitness value of 1 on the training case using the F1-Score as the cost function, as illustrated in Fig 5. All figures have demonstrated the fitness evolution over 50 epochs. In this case, each fold has upheld a steady fitness level during the feature selection procedure without major variations.

Table 11 represents the optimized hyperparameter settings for the CB classifier and the corresponding selected feature index (SFI) obtained using the SLOA optimizer across a custom 5-fold validation strategy. The optimization consistently converged in the same solution across all folds, and the parameter values are: iterations = 266, learning rate = 0.13605110592812045, tree depth = 8, and L2 regularization = 7.07838961573286. In addition, the SLOA optimizer has selected 19 features (SFI: 1, 3, 5, 7, 11, 12, 13, 14, 16, 17, 18, 20, 24, 25, 31, 32, 33, 35, 37) for each fold. However, the SLOA optimizer has selected the best features, which are associated with our target class. Although the dataset originally comprised 38 features, the SLOA_CB optimization process reduced this number and ultimately selected 19 informative features.

**Table 11:** CB Hyperparameter settings and selected feature indices for each fold using SLOA.

Fold No.	Hyperparameter and Selected Feature Index (SFI)
1	iterations: 266
	learning_rate: 0.13605110592812045
	depth: 8
	l2_leaf_reg: 7.107838961573286
	SFI: 1, 3, 5, 7, 11, 12, 13, 14, 16, 17, 18, 20, 24, 25, 31, 32, 33, 35, 37
2	iterations: 266
	learning_rate: 0.13605110592812045
	depth: 8
	l2_leaf_reg: 7.107838961573286
	SFI: 1, 3, 5, 7, 11, 12, 13, 14, 16, 17, 18, 20, 24, 25, 31, 32, 33, 35, 37
3	iterations: 266
	learning_rate: 0.13605110592812045
	depth: 8
	l2_leaf_reg: 7.107838961573286
	SFI: 1, 3, 5, 7, 11, 12, 13, 14, 16, 17, 18, 20, 24, 25, 31, 32, 33, 35, 37
4	iterations: 266
	learning_rate: 0.13605110592812045
	depth: 8
	l2_leaf_reg: 7.107838961573286
	SFI: 1, 3, 5, 7, 11, 12, 13, 14, 16, 17, 18, 20, 24, 25, 31, 32, 33, 35, 37
5	iterations: 266
	learning_rate: 0.13605110592812045
	depth: 8
	l2_leaf_reg: 7.107838961573286
	SFI: 1, 3, 5, 7, 11, 12, 13, 14, 16, 17, 18, 20, 24, 25, 31, 32, 33, 35, 37
Finalized	iterations: 266
	learning_rate: 0.13605110592812045
	depth: 8
	l2_leaf_reg: 7.107838961573286
	SFI: 1, 3, 5, 7, 11, 12, 13, 14, 16, 17, 18, 20, 24, 25, 31, 32, 33, 35, 37

#### 3.2.2 Performance evaluation.

Table 12 includes the results of the classification with the CB classifier model optimized with SLOA_CB. In all the training folds, the model has perfectly classified and achieved 100% with an accuracy, precision, recall, and F1-score, indicating that the model has been able to perfectly fit the training samples without misclassification.

**Table 12:** Model results using SLOA_CB.

Fold	Train				Test			
	Acc. (%)	F1-score. (%)	Pre. (%)	Rec. (%)	Acc. (%)	F1-score (%)	Pre. (%)	Rec. (%)
Fold-1	100	100	100	100	85.42	83.72	90.00	78.26
Fold-2	100	100	100	100	78.12	77.89	77.08	78.72
Fold-3	100	100	100	100	71.88	71.58	75.56	68.00
Fold-4	100	100	100	100	87.50	87.23	87.23	87.23
Fold-5	100	100	100	100	79.17	78.26	80.00	76.60
Mean	100	100	100	100	80.42	79.74	81.97	77.76

On the test sets, we have observed a slight variation in performance across the folds. Fold-4 has achieved the highest accuracy of 87.50%, with an F1-score of 87.23%, precision of 87.23%, and recall of 87.23%. This result has highlighted a balanced ratio between false positives and false negatives. Conversely, Fold-3 has obtained the poorest performance, which is 71.88% accuracy and an F1-score of 71.58%, which indicates the highest number of samples have been misclassified in this case.

In addition, the average test performance across all folds has obtained 80.42% accuracy, 79.74% F1-score, 81.97% precision, and 77.76% recall, which reflects that the proposed SLOA_CB approach effectively preserves the predictive power. The high precision value indicates that this model does a good job of reducing false positive cases (i.e., patients incorrectly classified as having gallstones), whereas the recall scores exhibit some variability, but are overall satisfactory with regard to correctly identifying positive gallstone cases.

#### 3.2.3 Confusion matrix analysis.

**Fig 6:**
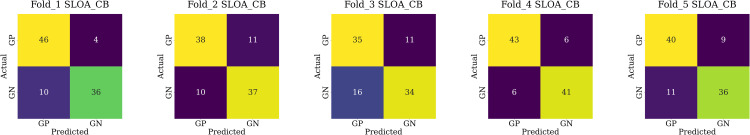
Each fold visual depiction of the confusion matrix of SLOA_CB.

The Fig 6 shows the confusion matrices of each fold of the proposed SLOA_CB model. The classes have been differentiated as GP and GN. In all folds, TP and TN (diagonal elements) consistently dominate, indicating that the model can correctly identify the majority of cases.

- Fold 1: Of 96 samples, 46 GP and 36 GN samples have been classified correctly, whereas 4 GP and 10 GN samples have been misclassified.
- Fold 2: The SLOA_CB has predicted 38 of the GP samples correctly and 37 of the GN samples correctly, whereas 11 GP and 10 GN samples have been misclassified.
- Fold 3: Out of the total samples, 35 and 34 have been correctly predicted as GP and GN, respectively, while 11 GP and 16 GN samples have been incorrectly predicted.
- Fold 4: In this case, the results have demonstrated improved performance, with 43 GP and 41 GN samples correctly classified, while 6 GP and 6 GN samples have been misclassified.
- Fold 5: The SLOA_CB has correctly identified 40 GP and 36 GN samples, while 9 GP and 11 GN samples have been mistakenly identified.

In this case, these findings have confirmed that the suggested approach has maintained a reasonable balance across folds, with evident capacity to differentiate between GP and GN cases. Although there has been slight variability in predictions across folds (e.g., Fold 3 has exhibited marginally higher misclassification rates), the fact that predictions have generally remained consistent has demonstrated the robustness and stability of SLOA_CB under custom cross-validation. Moreover, the model has been showing stable predictive behavior across different folds, which indicates its reliability for medical classification tasks.

#### 3.2.4 Misclassifications output of SLOA_CB.

Table 13 presents the detailed results of the misclassifications of each fold obtained using the SLOA_CB classifier. For each fold, the number and percentage of misclassified samples have been reported along with the exact indices of instances that are incorrectly predicted by the SLOA_CB classifier. In fold 1, the number of misclassified samples is 14, and the corresponding misclassification percentage is 14.58%. In addition, fold 2 has shown 21 misclassifications (21.88%). Similarly, folds 3,4, and 5 have contained the number of misclassified samples that are 27, 19, and 20, respectively, and the percentage of misclassifications is 28.12%, 12.50%, and 20.83%, respectively.

**Table 13:** Misclassified instances across all folds using the SLOA_CB classifier.

Fold No.	Number of Misclassified	Misclassification (%)	Misclassified Instances
1	14	14.58	73, 116, 118, 144, 176, 177, 194, 195, 203, 218, 220, 250, 299, 316
2	21	21.88	66, 69, 92, 95, 96, 115, 117, 125, 143, 148, 157, 172, 196, 199, 204, 206, 221, 226, 288, 295, 304
3	27	28.12	8, 43, 52, 91, 103, 105, 110, 128, 141, 153, 156, 178, 192, 200, 201, 207, 215, 216, 225, 227, 243, 251, 281, 291, 297, 309, 315
4	19	12.50	1, 3, 5, 7, 11, 12, 13, 14, 16, 17, 18, 20, 24, 25, 31, 32, 33, 35, 37
5	20	20.83	66, 69, 92, 94, 115, 117, 119, 148, 157, 163, 195, 196, 203, 206, 221, 288, 295, 304, 311, 316

#### 3.2.5 ROC curve analysis.

**Fig 7:**
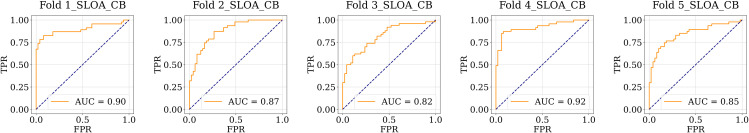
Each fold graphical representation of the ROC curve of SLOA_CB.

The ROC curves and resultant AUC values achieved on each fold during the custom 5-fold cross-validation of the proposed SLOA_CB model are reflected in Fig 7. The AUC statistics have been between 0.82 and 0.92, which demonstrates that the model has been stable with its good discriminative ability between GP and GN cases across folds. In particular, Fold 4 has shown the best AUC of 0.92, whereas Fold 3 is characterized by a slightly lower AUC of 0.82, which indicates certain variability between the fold sizes in terms of the classification abilities.

Overall, the model already has an average AUC of about 0.87, demonstrating that it is reliable and stable across unknown data. As presented in the curves, the TPR has been quite high compared to the FPR, which implies the model is sensitive and reliable in detecting GP patients. These results have indicated that the combination of SLOA and CatBoost has played a successful role in increasing the predictive accuracy without compromising the generalization performance across the folds.

#### 3.2.6 Controlling overfitting.

**Fig 8:**
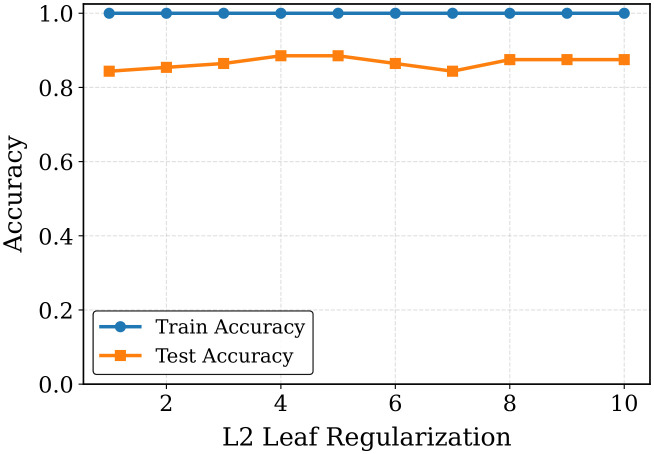
L2 leaf regularization vs accuracy gap of optimized CatBoost classifier.

The overfitting nature of the optimized CatBoost classifier is examined by assessing the performance of the model at varying L2 leaf regularization with the rest of the tuned hyperparameters held constant. The findings are provided in Table 14 and depicted in Fig 8. The accuracy of the training is also at 100% in all of the experimented regularization values, which implies that the model precisely fits the training data. Nevertheless, the testing accuracy ranges between 84.38% and 88.54%, indicating that the selection of an L2 leaf regularization parameter has an effect on model generalization. The least train-test gap (11.46%) is when the values of L2 leaf regularization are 4 and 5, and the test accuracy is the greatest (88.54%). This implies that moderate regularization is useful in regulating the complexity of the model and enhances the capability to generalize. Alternatively, high or low regularization values have the opposite effect since they result in a greater difference between the training and the performance with respect to testing and this implies a greater propensity to overfitting. On the whole, these findings indicate that the parameter of regularizing L2 leaves should be chosen to achieve a balance between model fitting and generalization in the CatBoost classifier.

**Table 14:** Performance of the optimized CatBoost varying L2 Leaf Regularization only for Fold 1.

L2 Leaf Reg	1	2	3	4	5	6	7	8	9	10
Training Accuracy (%)	100.0	100.0	100.0	100.0	100.0	100.0	100.0	100.0	100.0	100.0
Testing Accuracy (%)	84.38	85.42	86.46	88.54	88.54	86.46	84.38	87.50	87.50	87.50
Gap (%)	15.62	14.58	13.54	11.46	11.46	13.54	15.62	12.50	12.50	12.50

**Fig 9:**
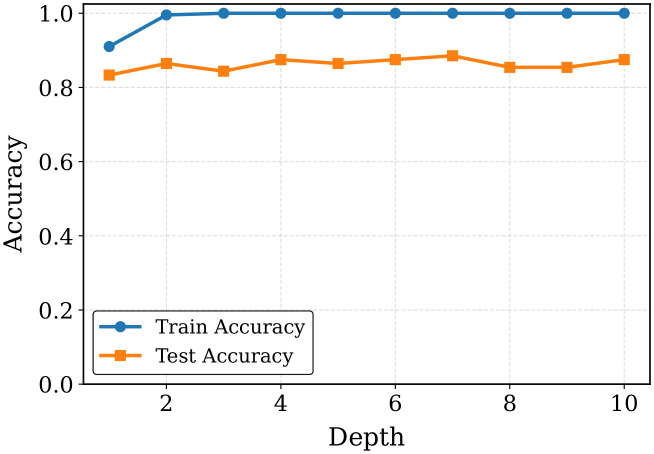
Tree depth vs accuracy gap of optimized CatBoost classifier.

Additionally, we have analyzed the results by varying the tree depth. As Fig 9 demonstrates, the training accuracy grows quickly and reaches 100% since depth 3, and shows that the model perfectly fits the training data. Nevertheless, the accuracy in testing also does not change much within the range, so it is around 84%−89% which indicates that the depth does not greatly improve the generalization performance any better. The accuracy gap between training and testing has been measured with the train-test gap as indicated in Table 15, with the gap growing up to 15.62% at depth 3, and this shows that overfitting had occurred. The reason behind this behavior is that more complex models can make the model more complex and enable the classifier to remember the training data instead of extrapolating to unseen samples. Of the tested tree depths, depth 7 has the best testing accuracy (88.54%) and lower train-test gap (11.46%), and is therefore a better balance of model complexity and generalization behavior. In general, the findings indicate that control of tree depth is significant towards reducing overfitting and ensuring stable predictive accuracy.

**Table 15:** Performance of the optimized CatBoost varying tree depth only for fold 1.

Tree Depth	1	2	3	4	5	6	7	8	9	10
Training Accuracy (%)	91.03	99.55	100.0	100.0	100.0	100.0	100.0	100.0	100.0	100.0
Testing Accuracy (%)	83.33	86.46	84.38	87.50	86.46	87.50	88.54	85.42	85.42	87.50
Gap (%)	7.70	13.09	15.62	12.50	13.54	12.50	11.46	14.58	14.58	12.50

According to the results, the CatBoost model is always perfect when it comes to training accuracy, whereas testing accuracy changes based on tree depth and L2 leaf regularization. The highest testing performance and smallest train-test gap (that is, better generalization) is obtained with moderate values of tree depth and L2 regularization (e.g., depth 7, L2 = 4–5). Generally, it is important to tune these hyperparameters carefully to ensure that the model is not too complex and also to prevent overfitting.

#### 3.2.7 SHAP analysis.

**Fig 10:**
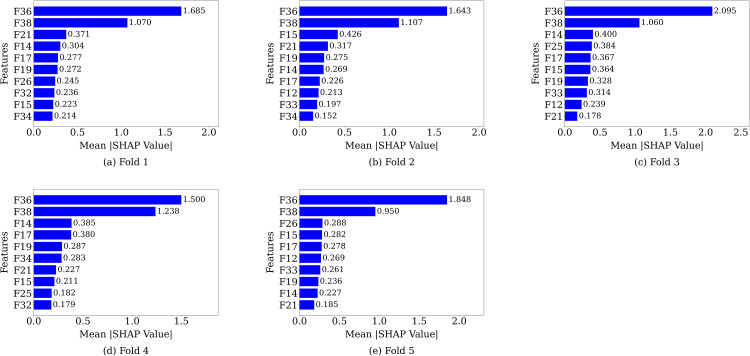
Mean absolute SHAP value of top 10 features for each fold using SLOA_CB classifier.

Mean absolute SHAP values of the top 10 features per fold for SLOA_CB are shown in Fig 10. In this figure, the horizontal bars show how much each feature contributes to the model’s predictions, with longer bars representing higher importance. In this case, the SHAP analysis has revealed consistent feature importance patterns for the SLOA_CB classifier at all folds. In all folds, features F36 (C-Reactive Protein) and F38 (Vitamin D) have demonstrated the highest mean absolute SHAP values, showing their dominant and stable contribution to the model predictions.

#### 3.2.8 LIME analysis.

**Fig 11:**
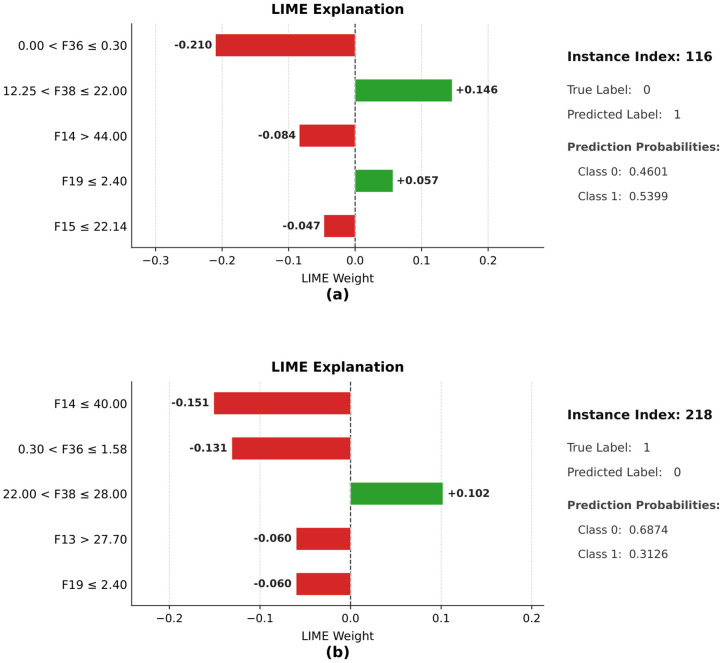
Visual representation of LIME explanations for misclassified instances (indices 116 and 218) using SLOA_CB.

Fig 11 shows the LIME of two misclassified examples (indexes 116 and 218) and indicates the local contribution of features that have affected the model in making the wrong predictions. In the case of index 116, the actual class is 0, the model has predicted the actual as 1 with the probability of 0.5399; the explanation has revealed that features F14, F15, and F38 have been contributive towards class 1, but features F19 and F36 have been contributive towards class 0.

The features F36 and F38 are the most influential and substantially higher among all the selected features, indicating strong and reliable predictive power. In addition, F14, F15, F17, and F19 have shown moderate SHAP values, implying that they are less influential compared to F36 and F38. Across all folds, fold 3 has achieved the highest mean absolute SHAP value of 2.095 for F36, emphasizing its strong effect. The minimum SHAP value observed for F36 is 1.500, recorded in fold 4, indicating a slightly reduced but still strong influence of this feature in that particular fold. In all folds, other relatively low SHAP values have indicated that the model is less dependent on them. Overall, the analysis has highlighted that the features F36 and F38 are the most influential features, and the consistent ranking across folds confirms the robustness and generalizability of the model.

The positive effect of F19 and F38 has, however, overridden the opposing contributions, resulting in a misclassification. In the case of index 218, the real class is 1, whereas the model has predicted it to be 0 with a probability of 0.6874; the LIME output has shown that F13, F14, F19, and F36 are some of the features that have played a strong role to predict 0, whereas the positive role played by F38 was not substantial enough to offset the overwhelming negative effects. In general, the explanations have shown that the misclassifications have been a result of competing local feature contributions whereby a small number of highly influential features have controlled the decision-making process, as such showing how sensitive the model is to local decision boundaries and the ability of LIME to diagnose the errors of prediction.

**Fig 12:**
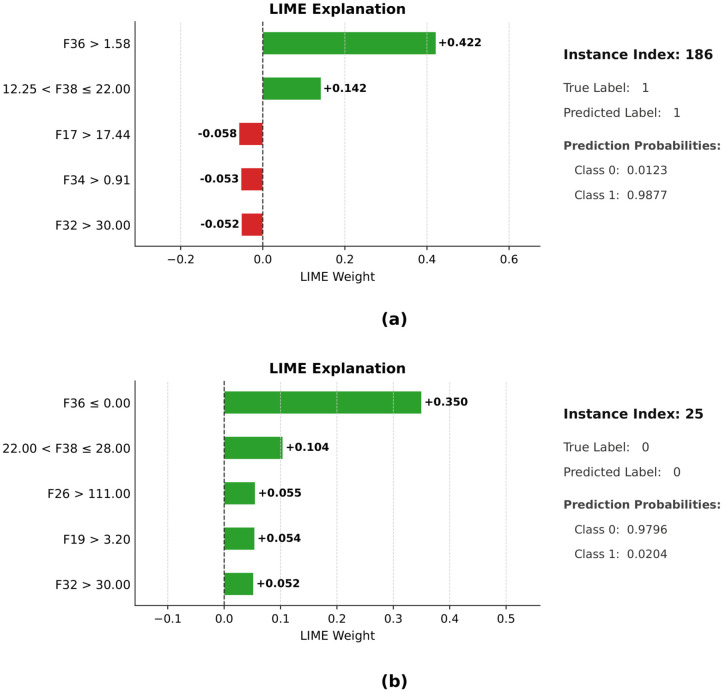
Visual representation of LIME explanations for correctly classified instances (indices 186 and 25) using SLOA_CB.

Fig 12 shows the LIME explanations of two correctly classified examples (index 186 and index 25) produced by the SLOA CB model. Instance index 186 shows that the features F17, F32, F34, F36, and F38 mainly influence the prediction, with F36 and F38 having a positive impact and F17, F32, and F34 having a negative impact on the prediction. Similarly, instance index 25 indicates that F19, F26, F32, F36, and F38 are the key features influencing the classification decision, and most of these features appear to favor the predicted class. The prediction summaries show that the true labels and predicted labels are the same in both cases, and the model has placed high probabilities on the corresponding predicted labels, which proves the reliability of the chosen features in the decision-making of the model.

#### 3.2.9 DiCE analysis.

CRP and Vitamin D are the top two features in all the developed frameworks based on feature selection, SHAP, and LIME analysis. These specific features have been analyzed through a DiCE analysis to gain a better insight into how they contribute to individual predictions. This section has presented counterfactual explanations for two misclassified instances (indices 116 and 118) using DiCE for the SLOA_CB model.

Table 16 shows five counterfactual explanations obtained with DiCE on the SLOA_CB model using instance index 116, which is GP (class 0) as the actual classification. In the initial case, CRP (F36) =0.18 and Vitamin D (F38) =13.7, and the model has predicted GS status to be 1 (GN), which is not the case. The counterfactual explanations demonstrate that the variations of Vitamin D have affected the model prediction with CRP kept constant at 0.18 in all cases. Vitamin D rose in CF-1 (13.7 → 36.4), CF-2 (23.7 → 36.0), CF-3 (34.8 → 36.0), CF-4 (32.1 → 34.8), and CF-5 (29.9 → 36.0) in each case, and the predicted class changed from 1 (GN) to 0 (GP) in all. This trend has shown that, Vitamin D has a significant and independent influence on the local decision boundary of the SLOA_CB model so that predictions can match the actual gallstone (GS) condition, whereas CRP has less influence on the decision.

**Table 16:** Five counterfactual explanations generated using DiCE for the SLOA_CB, demonstrating how variations in CRP (F36) and Vitamin D (F38) influence the Gallstone Status prediction for instance index 116.

Counterfactual No.	CRP	Vitamin D	Gallstone Status
1	0.18	13.7 → 36.4	1 → 0
2	0.18	13.7 → 23.7	1 → 0
3	0.18	13.7 → 36.0	1 → 0
4	0.18	13.7 → 34.8	1 → 0
5	0.18	13.7 → 29.9	1 → 0

Table 17 shows five counterfactual explanations that have been generated using DiCE for the SLOA_CB model, for instance index 218, whose actual class is GN (class 1). In the original instance, CRP (F36) = 0.5 and Vitamin D (F38) = 23.6, and the GS status has been incorrectly predicted as GP (class 0). In CF-1, decreases Vitamin D (23.6 → 11.6) while keeping CRP constant have resulted in the predicted class being changed to GN (class 1), suggesting an effect of Vitamin D. In CF-2, CF-2, and CF-3, Vitamin D has been held constant, while CRP has been increased to 11.0, 34.8, and 3.9, respectively, which alone has been sufficient to reverse the prediction, highlighting CRP as a highly influential feature. Finally, in CF-5, increases in CRP (0.5 → 13.3) and decreases in Vitamin D (23.6 → 19.5) have again yielded the correct prediction. Overall, the counterfactuals have consistently demonstrated that CRP and Vitamin D exert stronger effects for this instance, identifying them as the most significant features influencing the model’s decision boundary.

**Table 17:** Five counterfactual explanations generated using DiCE for the SLOA_CB, demonstrating how variations in CRP (F36) and Vitamin D (F38) influence the Gallstone Status prediction for instance index 218.

Counterfactual No.	CRP	Vitamin D	Gallstone Status
1	0.5	23.6 → 11.6	0 → 1
2	0.5 → 11.0	23.6	0 → 1
3	0.5 → 34.8	23.6	0 → 1
4	0.5 → 22.3	23.6	0 → 1
5	0.5 → 13.3	23.6 → 19.5	0 → 1

## 4. Discussion

In this study, a tabular dataset of gallstone (GS) disease is classified into gallstone-positive and gallstone-negative categories using machine learning (ML) techniques. Most existing machine learning studies on gallstone disease have primarily focused on image-based data, with comparatively limited work on tabular datasets. Table 18 displays a comparative analysis between our proposed approaches with existing works. Bozdag et al. [8] have indicated that the image-based accuracy of gallstone detection is 94.4%, and Wang et al. [9] have recorded an amazing AUC of 0.995. The image accuracy of 98% obtained by Obaid et al. [11] is the highest among the studies compared with the 86.5% accuracy in Pang et al. [10]. In case, with tabular data, Esen et al. [18] obtained 85.42% accuracy with the use of 38 clinical features, which proves that both structured and imaging data are capable of successfully aiding the prediction of gallstone diseases. Furthermore, Li et al. [21] achieved a test accuracy of 81.25% by using a stacking ensemble of Random Forest, XGBoost, and Support Vector Machine as base learners with the meta-classifier of Logistic Regression.

**Table 18:** Comparison of existing studies and proposed frameworks for gallstone disease.

Serial No.	Author / Framework	Dataset	Performance
01	Bozdag et al. [8]	Image	Accuracy = 94.4%
02	Wang et al. [9]	Image	AUC = 0.995
03	Obaid et al. [11]	Image	Accuracy = 98%
04	Pang et al. [10]	Image	Accuracy = 86.5%
05	Esen et al. [20]	Tabular	Accuracy = 85.42%; Features = 38
06	Li et al. [21]	Tabular	Accuracy = 81.25%;
**Our Proposed Frameworks**			
01	CB with CV (Mean)	Tabular	Accuracy = 79.58%, F1-score = 79.01%
			Precision = 80.91%, Recall = 77.36%; Features = 38
02	CB with CV (Fold-1)	Tabular	Accuracy = 86.46%, F1-score = 85.39%
			Precision = 88.37%, Recall = 82.61%; Features = 38
03	SLOA_CB with CV (Mean)	Tabular	Accuracy = 80.42%, F1-score = 79.94%
			Precision = 81.97%, Recall = 77.76%; Features = 19
04	SLOA_CB with CV (Fold-4)	Tabular	Accuracy = 87.50%, F1-score = 87.23%
			Precision = 87.23%, Recall = 87.23%; Features = 19

Comparatively, the approaches that we have proposed in this study include CB with CV (Cross-Validation) and SLOA_CB with CV. Here, a 5-fold CV with CB model methods has achieved a mean accuracy of 79.58%, a mean F1-score of 79.01%, a mean precision of 80.91%, and a mean recall of 77.36% when using a total of 38 features. However, in this case, fold-1 has attained an accuracy of 86.46%, F1-score of 85.39%, precision of 88.37%, and recall of 82.61% in 5-fold CV with CB model. This result indicates that the CB model is capable of reliably distinguishing between gallstone-positive and gallstone-negative cases, which is critical for clinical decision-making. In addition, these results also convey that the CB model generalizes well to unseen data and exhibits stable performance across the validation folds. Additionally, utilizing the 19 selected features, a 5-fold CV with the SLOA_CB model achieved a mean accuracy of 80.42%, a mean F1-score of 79.94%, a mean precision of 81.97%, and a mean recall of 77.76%. In this case, an accuracy of 87.50%, along with F1-score, precision, and recall values that are 87.23%, has been achieved in fold-4 using 5-fold CV with SLOA_CB model, indicating balanced classification performance. As F1-score, precision, and recall values are all 87.23%, suggesting that the SLOA_CB model has neither strongly favored false positives nor false negatives in fold-4. Overall, these results indicate that the proposed model aligns well with existing gallstone prediction approaches. Moreover, in the context of model explainability, SHAP, LIME, and DiCE have been applied, and in each case, CRP (F36), Vitamin D (F38), and AST (F31) have been identified as the most significant features for the gallstone disease prediction. In particular, CRP is linked to inflammation related to gallstones and the severity of the disease, making it a valuable biomarker for gallbladder disorders [47]. In a separate medical study involving 4484 participants, it was discovered that higher CRP levels correlate with an increased occurrence of gallstones [48]. Liu et al. have stated that CRP functions as an independent risk factor for gallstone disease [49]. A different study examining 6873 individuals indicated that greater intake of dietary vitamin D (D2 + D3) is positively correlated with the incidence of gallstones [50]. Therefore, prior research has established the connections between CRP and Vitamin D with the classification of gallstones. Our research has also identified these two features as the most significant ones.

### 4.1 Future studies

This research has effectively classified the presence of gallstones using a tabular dataset and has shown a good result with CB, with CV, and SLOA_CB with CV. In addition to effective prediction, it has offered insights into the importance of features, reasons why it misclassified, and correction measures, which can be used in clinical practice. Future studies must aim at the expansion of data and external validation of the models, and apply the framework to the smart diagnostic system or mobile health app to improve the aspects of early detection, decision-making, and patient care. In addition, the further work will involve the assessment of the model on the multi-center data to warrant its clinical reliability among the various population groups.

### 4.2 Limitations

This research has a number of limitations that need to be considered. Firstly, the sample size is limited (319 individuals), which lowers the results’ statistical power and dependability. Secondly, the dataset has been used in this study, which has originated from an Internal Medicine Outpatient Clinic of Ankara VM Medical Park Hospital. Therefore, the results may not be broadly relevant to more diverse groups with different demographic and clinical backgrounds because the data were collected from a single center. Thirdly, external validation—which is essential for confirming the robustness and clinical applicability of the models—was hampered by the absence of an independent dataset with similar properties. Finally, a discrepancy between training and testing performance was observed, suggesting a potential risk of overfitting, perhaps due to the small sample size. Larger, multi-center datasets with external validation should be the goal of future studies in order to increase the resilience and application of gallstone prediction models.

## 5. Conclusion

Gallstones are solid formations that develop in the gallbladder from accumulated bile substances, and the majority of individuals with gallstones (around 80%) do not exhibit any symptoms [51,52]. According to recent research, machine learning methods for gallstone prediction employing both image and tabular datasets are becoming more and more popular. The CatBoost classifier model and the Sea Lion Optimization algorithm have been employed in this study. In this study, the main approaches investigated are CatBoost with cross-validation and CatBoost optimized using the Sea Lion Optimization (SLO) algorithm with cross-validation. Using a total of 38 features, the CB classifier model techniques with 5-fold cross-validation have achieved a mean accuracy of 79.58%, a mean F1 score of 79.01%, a mean precision of 80.91%, and a mean recall of 77.36%. In the 5-fold cross-validation utilizing the CB classifier model, fold-1 specifically has attained an accuracy of 86.46%, an F1 score of 85.39%, a precision rate of 88.37%, and a recall of 82.61%. Additionally, using the 19 selected features, the SLOA_CB model applied in 5-fold cross-validation achieved a mean accuracy of 80.42%, a mean F1 score of 79.94%, a mean precision of 81.97%, and a mean recall of 77.76%. In this case, fold-4 has demonstrated a well-balanced classification performance with an accuracy of 87.50% and an F1 score, precision, and recall of 87.23%. Finally, in the realm of model explainability, SHAP, LIME, and DiCE have been utilized, with C-Reactive Protein (CRP) (F36) and Vitamin D (F38)recognized as the key features impacting the prediction of gallstone disease in every instance.
